# Family SES amplifies genetic influences on organized activity involvement from childhood to young adulthood: evidence from the German twin family panel

**DOI:** 10.3389/fsoc.2026.1780253

**Published:** 2026-06-30

**Authors:** Yixuan Liu

**Affiliations:** Faculty of Sociology, Bielefeld University, Bielefeld, Germany

**Keywords:** family socioeconomic status (SES), gene–environment interaction, German twin family panel (TwinLife), organized activity involvement, social inequalities

## Abstract

Organized activity involvement contributes to social cohesion and individual development, yet substantial socioeconomic disparities persist. Although prior research shows that both genetic differences and family socioeconomic status (SES) shape organized activity involvement, less is known about how socioeconomic contexts condition the realization of genetic potential. Drawing on gene–environment interaction theory, this study examines whether family SES amplifies or attenuates genetic influences on organized activity involvement. We contrast two competing perspectives: the High-SES Amplification model, which predicts stronger genetic influences in advantaged contexts, and the Low-SES Expression model, which predicts stronger genetic influences in disadvantaged contexts. Using data from the German Twin Family Panel (TwinLife, ages 4–25, *N* = 7,973), we estimate classical twin models that decompose variance in organized activity involvement into additive genetic, shared environmental, and non-shared environmental components and test whether these components vary across SES levels. Results show that genetic influences are stronger at higher levels of family SES, supporting the High-SES Amplification hypothesis and highlighting how family SES shapes inequality in organized activity involvement by moderating the realization of genetic potential.

## 1 Introduction

Organized activity involvement plays an important role in fostering social cohesion and sustaining democratic institutions (Putnam, 2001; de Tocqueville, 2002), and it is consistently associated with individual well-being and developmental outcomes across the life course (Morris, 2015; Ennis and Tonkin, 2018; Zarrett et al., 2021; Oubiña López and Gómez Baya, 2025). Organized activity involvement refers to participation in structured, organized group activities, including sports clubs, choir or theater groups, religious groups, volunteer organizations, political organizations, and other clubs or associations (Rauschenbach and Bien, 2012). Further, the unequal distribution of organized activity involvement across social groups reflects differences in individual potential as well as unequal constraints and opportunities individuals encounter in their social environments. Genes constitute one source of individual potential, yet whether and to what extent this potential is realized depends on environmental conditions. Understanding how the realization of genetic potential differs across social groups—for example, by family socioeconomic status (SES)—therefore speaks to a core sociological concern with inequality.

**Figure 1:**
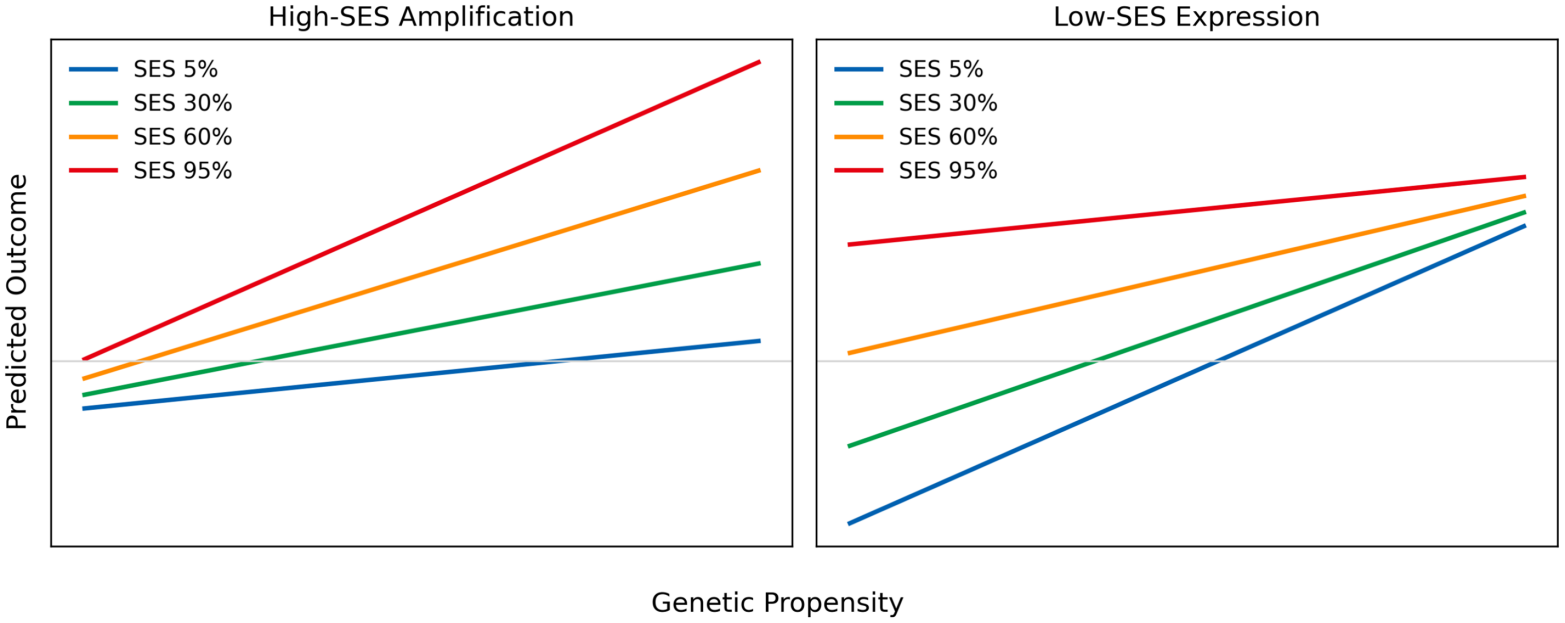
Conceptual illustration of genetic influences on outcomes across SES. Assume SES and genetic propensity positively predict outcome; colored lines represent different SES quantiles (5%, 30%, 60%, 95%).

Previous gene–environment interaction (G × E) research examines how social contexts condition the extent to which genetic differences are realized. This study builds on two theoretical models in this literature (Bronfenbrenner and Ceci, 1994; Shanahan and Hofer, 2005; Saunders, 2010; Nielsen, 2016; Lin, 2020). The High-SES Amplification model posits that genetic influences are more pronounced in advantaged environments, where abundant resources allow individual potential to be fully realized. In contrast, the Low-SES Expression model argues that genetic influences are stronger in disadvantaged contexts, where structural constraints limit opportunities such that only individuals with high genetic potential are able to succeed. Figure 1 illustrates how these models generate distinct predictions about the relationship between genetic propensity and outcomes across levels of SES. In the absence of gene–environment interaction, genetic influences would remain constant across SES.

Prior empirical research on organized activity involvement provides support for both perspectives, depending on how opportunities and constraints operate. Organized activity involvement typically requires financial resources, parental support, and institutional access (Covay and Carbonaro, 2010; Lareau, 2011; Snellman et al., 2015). In higher-SES contexts, the widespread availability of resources may allow genetic differences to manifest more fully by lowering structural barriers. At the same time, when organized activity involvement is institutionalized as a normative component of advantaged family life, it may be less contingent on individual potential, thereby attenuated genetic influences. In contrast, in lower-SES contexts where opportunities are scarce and costs are higher, structural constraints may restrict organized activity involvement to individuals whose potential is sufficiently strong to sustain engagement despite adverse conditions.

To test these two competing hypotheses, we draw on data from the German Twin Family Panel (TwinLife) and estimate a series of structural equation models using the classical twin design. This approach decomposes variance in organized activity involvement into additive genetic (A), shared environmental (C), and non-shared environmental (E) components. We then extend these models by incorporating family SES as a moderator, allowing genetic and environmental variance components to vary systematically across levels of SES. This modeling strategy enables a direct test of whether the influence of genetic differences on organized activity involvement is amplified or attenuated under conditions of socioeconomic advantage or disadvantage.

## 2 Data and methods

### 2.1 Data

We used data from the German Twin Family Panel (TwinLife), a nationally representative, cohort-sequential study comprising four cohorts of families with same-sex twins in Germany who were 5 (cohort 1), 11 (cohort 2), 17 (cohort 3), and 22–24 (cohort 4) years old at the first face-to-face (F2F1) interview in 2014 or 2015 (Mönkediek et al., 2019; Diewald et al., 2024). TwinLife applied a register-based probability sampling design and includes families across the entire socioeconomic spectrum in Germany (Lang and Kottwitz, 2020). The TwinLife study was reviewed and approved by the German Psychological Society (protocol number: RR 11.2009).

The field period for F2F1 was from September 28, 2014, to May 28, 2015 (Subsample A), and from September 16, 2015, to April 18, 2016 (Subsample B). At F2F1, there were 8,192 twins from 4,096 families, forming 4,096 complete twin pairs. Specifically, there were 2018 twins in cohort 1, 2086 in cohort 2, 2,122 in cohort 3, and 1966 in cohort 4.

For family-level information, among the 4,096 families, 3 had missing zygosity, and 7 had missing family SES. For individual-level information, among the 8,192 twins, 199 had no valid measure of organized activity involvement. We excluded 20 families that were missing either zygosity or family SES, and we excluded 71 families with no valid measure of organized activity involvement for either twin. The final analytic sample included 7,973 twins from 4,015 families: 3735 monozygotic (MZ) twins, 4,238 dizygotic (DZ) twins, 3,609 males and 4,364 females, and 1934 twins in cohort 1, 2013 in cohort 2, 2091 in cohort 3, and 1935 in cohort 4.

### 2.2 Measures

#### 2.2.1 Organized activity involvement

Organized activity involvement was measured using items asking how often twins engaged in organized activities (Rauschenbach and Bien, 2012). The activities included: (1) sports clubs, (2) choir or theater groups, (3) church or religious groups, (4) volunteer fire departments or technical assistance organizations, (5) local clubs or marksmen’s associations, (6) trade unions, occupational associations, or student councils, (7) political organizations, parties, or citizens’ initiatives, and (8) other clubs or associations. Two items were asked only of twins in cohorts 2, 3, and 4 [(5) local clubs or marksmen’s associations; (6) trade unions, occupational associations, or student councils], and one item was asked only of twins in cohorts 3 and 4 [(7) political organizations, parties, or citizens’ initiatives].

Each item was rated on a four-point scale (1 = every week, 2 = every month, 3 = less than once a month, 4 = never). For participants aged 10 years and older, self-reported answer was used whenever available and supplemented with parent reports when self-reports were missing. For younger participants who did not complete the self-report, parent-reported information was used. Response categories were recoded into approximate monthly frequencies: 0 (“never”), 0.5 (“less than once a month”), 1 (“every month”), and 4 (“every week”). Responses across all activity domains were summarized to create a continuous index, with higher values indicating greater overall organized activity involvement. Table 1 displays the item-level summary statistics for the organized activity involvement items. The sports clubs was the most prevalent activity reported.

**Table 1:** Item-level summary statistics for organized activity involvement.

Characteristics	2009/2010 N = 2,018	2003/2004 N = 2,086	1997/1998 N = 2,122	1990–1993 N = 1,966
Sports	2.51 (1.90)	2.81 (1.79)	2.21 (1.95)	1.60 (1.90)
Missing	85	97	88	64
Choir/theater	1.19 (1.79)	1.45 (1.88)	0.72 (1.51)	0.27 (0.95)
Missing	127	150	114	112
Church/religious	0.38 (1.02)	0.62 (1.30)	0.47 (1.16)	0.27 (0.89)
Missing	135	159	118	111
Volunteer fire/technical assistance	0.04 (0.37)	0.15 (0.74)	0.13 (0.69)	0.10 (0.57)
Missing	162	186	114	116
Local/marksmen	0.00 (0.00)	0.06 (0.43)	0.07 (0.49)	0.04 (0.32)
Missing	2,015	191	124	117
Trade/occupational/student councils	0.00 (0.00)	0.19 (0.68)	0.19 (0.72)	0.09 (0.45)
Missing	2,015	189	157	126
Political/party/citizens’ initiative	NA (NA)	NA (NA)	0.03 (0.30)	0.05 (0.36)
Missing	2,018	2,086	124	114
Other	0.23 (0.91)	0.26 (0.95)	0.28 (0.95)	0.26 (0.90)
Missing	197	255	211	157

Mean (SD); missing = number of observations with missing data; NA = not available (missing data). N twins = 8,192 and N families = 4,096. Data source: German Twin Family Panel (TwinLife).

8,192 cases provided at least one valid response, 0 cases with all items missing were excluded, and partially missing items were ignored and contributed as zero to the final sum. To reduce the influence of extreme values on the analysis of Organized Activity Involvement without removing data, we applied winsorization. Using Interquartile Range (IQR)-based thresholds, values below the lower extreme bound or above the upper extreme bound were replaced with the corresponding bounds, while values equal to the bounds remained unchanged. We capped 2 extreme observations beyond 3 × IQR.

#### 2.2.2 Family SES

Family SES was measured as a composite factor based on the mean level of parental years of education, occupational status (International Socio-Economic Index, ISEI), and household net income. Parental years of education, occupational status and household net income were adjusted for age to account for cohort-related differences before creating the factor. Household net income was capped at 3 × IQR for 98 families. A latent family SES score was constructed using confirmatory factor analysis (CFA) with the lavaan package (Rosseel et al., 2012; Rosseel, 2012). 7 rows consisting entirely of missing values were excluded, and partial missingness was handled using full information maximum likelihood (FIML) (Enders, 2010). Internal consistency was high ( α = 0.777), with higher factor scores indicating higher levels of family SES. We confirmed that 0 SES value exceeded 3 × IQR.

#### 2.2.3 Zygosity, sex and age

Twin zygosity for same-sex twin pairs was determined using questionnaires and validated with DNA subsamples; see the TwinLife technical report for details (Lenau et al., 2017). Monozygotic (MZ) twins share 100% of segregating alleles, whereas dizygotic (DZ) twins share, on average, 50%. Sex was recorded via report. For zygosity, MZ twins were coded as 0 and DZ twins as 1. For sex, males were coded as 0 and females as 1. Age was measured in years (to the nearest month) at the time of the family questionnaire.

### 2.3 Analyses

#### 2.3.1 Summary statistics

Table 2 displays the summary statistics by birth cohorts (cohort 1: 2009/2010, average age 5; cohort 2: 2003/2004, average age 11; cohort 3: 1997/1998, average age 17; and cohort 4: 1990–1993, average age 23) and for the total sample. Means and standard deviations (SD) are reported for continuous variables, and numbers and percentages [N (%)] are reported for categorical variables. The monthly frequency of organized activity involvement varied across cohorts, with the highest mean reported in cohort 2 [2003/2004; Mean (SD) = 5.36 (3.45)] and the lowest in cohort 4 [1990–1993; Mean (SD) = 2.61 (2.74)]. Missing data for this variable were modest across cohorts. Family SES was approximately centered around zero in the total sample [Mean (SD) = 0.00 (0.80)], with minor variations across cohorts. Missing SES data were minimal. Sex distribution was balanced within each cohort, with slightly more females overall [N (%) = 4,484 (55%)] than males [N (%) = 3,708 (45%)]. Among twins, the overall proportion of dizygotic twins [N (%) = 4,364 (53%)] was slightly higher than that of monozygotic twins [N (%) = 3,822 (47%)], with small amounts of missing data.

**Table 2:** Summary statistics by birth cohorts and for the total sample.

Characteristics	2009/2010 N = 2,018	2003/2004 N = 2,086	1997/1998 N = 2,122	1990–1993 N = 1,966	Total
Organized activity involvement	4.30 (3.38)	5.36 (3.45)	3.92 (3.47)	2.61 (2.74)	4.06 (3.42)
Missing	82	69	25	23	199
Family SES	0.20 (0.83)	0.09 (0.79)	−0.07 (0.77)	−0.23 (0.73)	0.00 (0.80)
Missing	0	2	4	8	14
Sex					
Male	980 (49%)	1,000 (48%)	906 (43%)	822 (42%)	3,708 (45%)
Female	1,038 (51%)	1,086 (52%)	1,216 (57%)	1,144 (58%)	4,484 (55%)
Age	5.0 (0.4)	11.0 (0.3)	17.0 (0.3)	23.0 (0.8)	14.0 (6.7)
Zygosity					
Monozygotic	868 (43%)	864 (41%)	1,018 (48%)	1,072 (55%)	3,822 (47%)
Dizygotic	1,148 (57%)	1,220 (59%)	1,102 (52%)	894 (45%)	4,364 (53%)
Missing	2	2	2	0	6

Mean (SD); N (%); N twins = 8,192 and N families = 4,096. Data source: German twin family panel (TwinLife).

#### 2.3.2 Correlations of monozygotic and dizygotic twins to guide model selection

First, we calculated correlations for monozygotic (MZ) and dizygotic (DZ) twins separately to guide model selection. The variance of a trait can be decomposed into four components: additive genetic influences (A); non-additive genetic influences (D), which include dominance and epistasis; shared environmental influences (C); and non-shared environmental influences (E), including measurement error. In the classical twin model, only three variance components can be estimated simultaneously; therefore, either the non-additive genetic component (D) or the shared environmental component (C) must be fixed to zero.

If the DZ twin correlation is less than half the MZ twin correlation (rDZ < 0.5 × rMZ), an ADE model is appropriate; otherwise, an ACE model is preferred (Knopik et al., 2017). We adjusted for age and sex before computing the twin correlations. Based on the total sample, the correlation for MZ twins (rMZ) is 0.79 (95% CI [0.781, 0.805]), and for DZ twins (rDZ) it is 0.68 (95% CI [0.664, 0.697]). Given that DZ twin correlations are not smaller than half the MZ twin correlations, the ACE model is preferred. The twin correlations by cohorts are shown in the Supplementary Table S1.

#### 2.3.3 ACE univariate variance decomposition

Second, we fit a series of univariate variance decomposition models as structural equation models with the ACE components as latent factors with variances fixed to 1 and path coefficients estimated freely (Neale and Maes, 2004). Sex and chronological age were adjusted for as covariates to avoid overestimating shared environmental influences (McGue and Bouchard, 1984). Missing data were handled using full information maximum likelihood (FIML) (Enders, 2010).

We first fit the ACE model and all of its nested models. Nested models were compared using chi-square difference tests. If a nested model with fewer parameters did not significantly worsen the fit compared with the expanded model, we preferred the parsimonious model; otherwise, the expanded ACE model was retained. Non-nested models were compared using AIC, with lower values indicating better fit. The variance decomposition model is:


$$Y=mu_{1}+\mathrm{bco}v_{11}\cdot \mathrm{sex}+\mathrm{bco}v_{22}\cdot \mathrm{age}+a_{11}A+c_{11}C+e_{11}E$$

Here, $mu_{1}$ denotes the intercept. The coefficients $\mathrm{bco}v_{11}$ and $\mathrm{bco}v_{22}$ correspond to the covariates sex and age , respectively. The term $a_{11}A+c_{11}C+e_{11}E$ captures the residual variance after adjusting for covariates, where $a_{11}$ , $c_{11}$ , and $e_{11}$ are the path coefficients for the additive genetic (A), shared environmental (C), and non-shared environmental (E) components. These path coefficients represent the magnitude of the corresponding A, C, and E component influences.

#### 2.3.4 ACE univariate variance decomposition with moderator

Third, we fit moderated univariate variance decomposition models (Purcell, 2002). We added linear continuous moderation to the means and ACE components stepwise to assess whether the moderator influenced any variance components. Using the same model comparison procedures described above, we identified the best-fitting model.


$$\begin{array}{r} Y=\mu_{1}+\mathrm{bco}v_{11}\cdot \mathrm{sex}+\mathrm{bco}v_{22}\cdot \mathrm{age}+\text{bMod}M_{11}\cdot \mathrm{mod}\\ +(a_{11}+\mathrm{bm}1a_{11}\cdot \mathrm{mod})\;A+(c_{11}+\mathrm{bm}1c_{11}\cdot \mathrm{mod})\;C+(e_{11}+\mathrm{bm}1e_{11}\cdot \mathrm{mod})\;E \end{array}$$

Here, $mu_{1}$ denotes the intercept; $\mathrm{bco}v_{11}$ and $\mathrm{bco}v_{22}$ correspond to the covariates sex and age , respectively; and $\text{bMod}M_{11}$ represents the influence of the moderator on the mean. The expressions $(a_{11}+\mathrm{bm}1a_{11}\;\mathrm{mod})A$ , $(c_{11}+\mathrm{bm}1c_{11}\;\mathrm{mod})C$ , and $(e_{11}+\mathrm{bm}1e_{11}\;\mathrm{mod})E$ model the moderation influences of the additive genetic (A), shared environmental (C), and non-shared environmental (E) components by family SES. That is, we directly tested whether the magnitude of the A, C, or E influences changes across levels of family SES.

Analyses were conducted in R 4.5.2 (R Core Team, 2025). Data were imputed, and correlations were computed using the tidyverse (Wickham et al., 2019) and the easystats framework (Lüdecke et al., 2022). Summary statistics were generated with the gtsummary package (Sjoberg et al., 2021). Structural equation models were fitted using OpenMx (Neale et al., 2016) and its wrapper twinflex (Ruks, 2025).

#### 2.3.5 Sensitivity analyses

To explore potential developmental stage differences in genetic and environmental influences, we first tested whether age moderates the influence of A, C, and E on organized activity involvement. Second, we examined whether and how family SES and age together moderate the influences of ACE components, allowing us to assess whether the pattern of family SES moderation varies across age.

## 3 Results

### 3.1 Univariate variance decomposition models of organized activity involvement

Table 3 displays the four models decomposing organized activity involvement into A, C, and E components. Model 1 presents the path coefficients of the best-fitting model (ACE). The model comparison results are shown in Supplementary Table S2. There was no evidence that females and males differed significantly in organized activity involvement, and age was significantly negatively associated with organized activity involvement. All ACE components significantly positively influenced organized activity involvement.

**Table 3:** Decomposition of organized activity involvement into ACE components.

Parameter	Model 1	Model 2	Model 3	Model 4
A	0.439***		0.844***	
	(0.026)		(0.009)	
C	0.749***	0.839***		
	(0.017)	(0.011)		
E	0.45***	0.503***	0.426***	0.978***
	(0.007)	(0.006)	(0.006)	(0.008)
Sex	−0.027	−0.026	−0.028	−0.024
	(0.029)	(0.029)	(0.027)	(0.022)
Age	−0.21***	−0.209***	−0.212***	−0.209***
	(0.014)	(0.014)	(0.013)	(0.011)
AIC	19127.15	19200.38	19508.55	22273.28

Standard errors are in parentheses; *p < 0.05, **p < 0.01, ***p < 0.001; organized activity involvement and age are standardized; sex (0 = male, 1 = female); the intercept is omitted; N twins = 7,973 and N families = 4,015. Data source: German Twin Family Panel (TwinLife).

**Figure 2:**
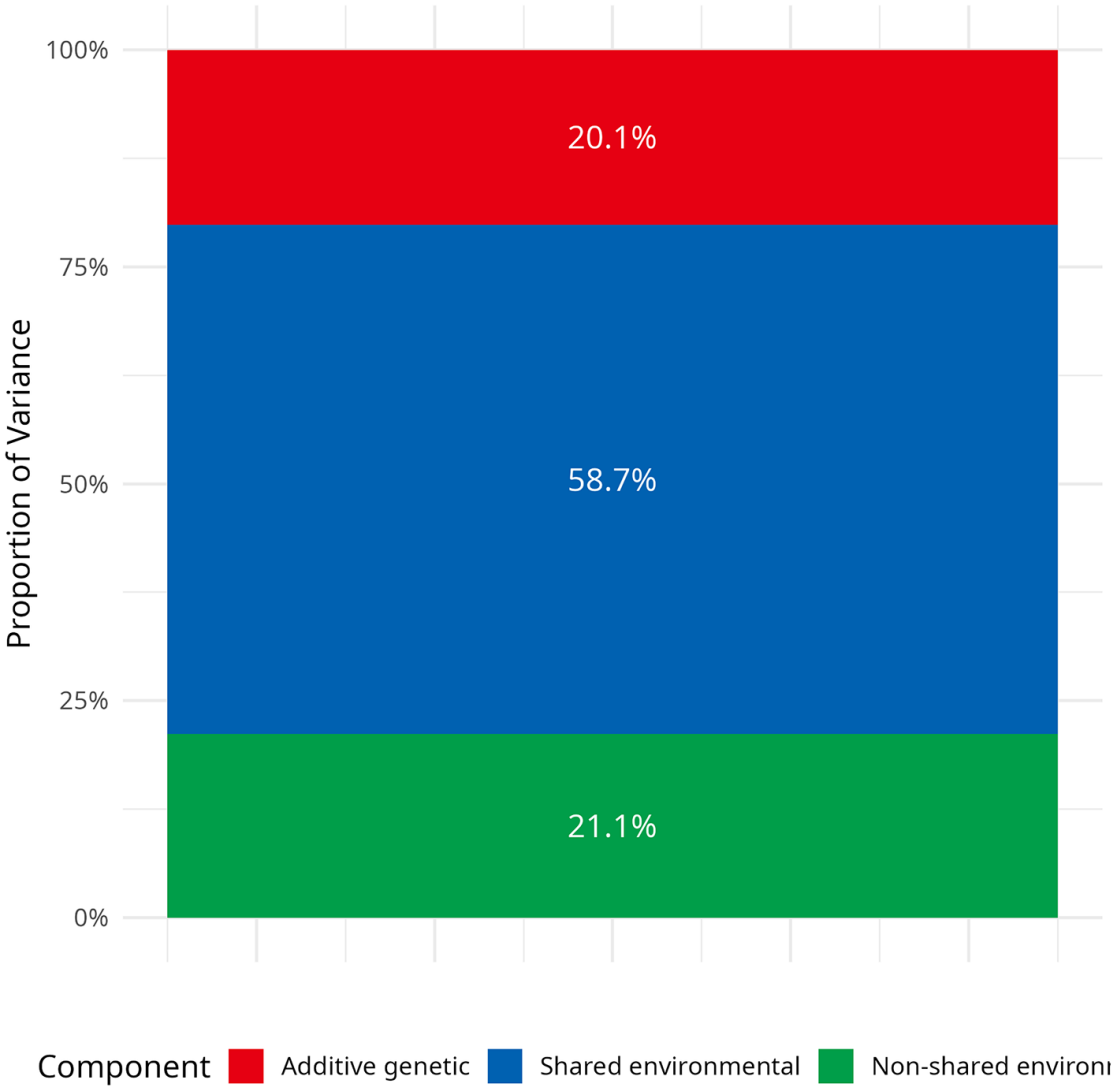
Proportions of variance explained by the ACE components. The proportions of variance were calculated as the squared path coefficients divided by the sum of the squared path coefficients of the ACE components in the best-fitting model. Sex and age were adjusted for in the model. Data source: German Twin Family Panel (TwinLife).

Figure 2 illustrates the proportions of variance explained by the ACE components based on the best-fitting model.

### 3.2 Moderated univariate variance decomposition models of organized activity involvement

Table 4 displays the best-fitting model decomposing organized activity involvement with moderation by family SES. The model comparison results are shown in the Supplementary Table S3. Considering both the chi-square difference tests and AIC, the model with A × family SES and C × family SES (ACEMMAMCM) provided the best fit to the data. There was no evidence that females and males differed significantly in organized activity involvement, and age was significantly negatively associated with organized activity involvement. Family SES was significantly positively associated with organized activity involvement. All ACE components significantly positively influenced organized activity involvement. Consistent with the High-SES Amplification hypothesis, the influence of A component was positively moderated by family SES: among individuals with high family SES, the genetic influences on organized activity involvement are stronger than among those with lower family SES. At the lowest family SES, the coefficient was 0.34, and at the highest family SES, 0.55.

**Table 4:** Decomposition of organized activity involvement into ACE components by family SES.

Parameter	Model 1
A	0.438***
	(0.026)
C	0.699***
	(0.018)
E	0.449***
	(0.007)
Sex	−0.020
	(0.028)
Age	−0.148***
	(0.014)
Family SES	0.279***
	(0.014)
A × family SES	0.043*
	(0.020)
C × family SES	0.043**
	(0.017)

Standard errors are in parentheses; *p < 0.05, **p < 0.01, ***p < 0.001; organized activity involvement, age and family SES are standardized; sex (0 = male, 1 = female); intercept is omitted; N twins = 7,973 and N families = 4,015. Data source: German Twin Family Panel (TwinLife).

**Figure 3:**
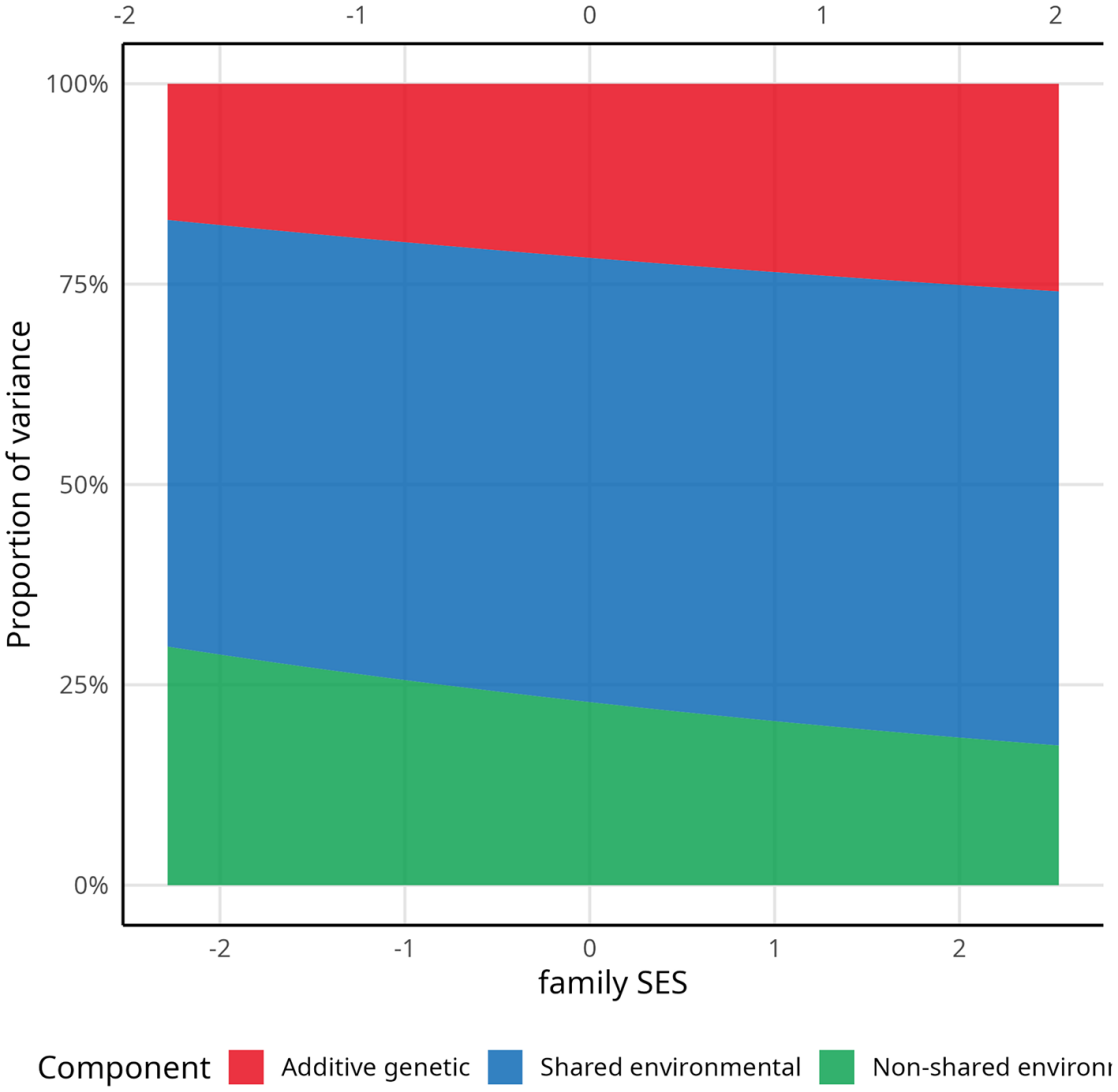
Proportions of variance explained by the ACE components. The proportions of variance were calculated as the squared path coefficients divided by the sum of the squared path coefficients of the ACE components in the best-fitting model. Sex, age and family SES were adjusted for in the model. Data source: German Twin Family Panel (TwinLife).

Figure 3 illustrates the proportions of variance explained by ACE components across different levels of family SES based on the best-fitting model. At the lowest family SES, the proportion of variance explained by A component was 0.17, and at the highest family SES, it was 0.26.

### 3.3 Sensitivity analyses

#### 3.3.1 Influences of ACE components by age

Based on the comparison results of nested age moderated models on organized activity involvement, and considering both the chi-square difference tests and AIC, the model with age moderation for all three ACE components provided the best fit to the data. Table 5 presents the best-fitting model decomposing organized activity involvement with moderation by age. Age moderated all three ACE components: the moderation influences were positive for A and E, and negative for C. Specifically, the coefficients of A and E increased across ages 4 to 25 (A: from 0.27 to 0.56; E: from 0.13 to 0.74), whereas the coefficient of C decreased over the same age range (from 1.12 to 0.31). These results reveal a developmental pattern: the influences of additive genetic (A) and non-shared environmental (E) increase with age, whereas shared environmental (C) influences decrease.

**Table 5:** Decomposition of organized activity involvement into ACE components by age.

Parameter	Model 1
A	0.409***
	(0.020)
C	0.729***
	(0.017)
E	0.422***
	(0.007)
Sex	−0.053
	(0.029)
Age	−0.247***
	(0.015)
A × age	0.095***
	(0.015)
C × age	−0.257***
	(0.018)
E × age	0.193***
	(0.006)

Standard errors are in parentheses; *p < 0.05, **p < 0.01, ***p < 0.001; organized activity involvement and age are standardized; sex (0 = male, 1 = female); intercept is omitted; N twins = 7,987 and N families = 4,022. Data source: German Twin Family Panel (TwinLife).

#### 3.3.2 Influences of ACE components by the interaction of age and family SES

Given that the influences of ACE components on organized activity involvement change across age, we next examined whether the interaction of age and family SES moderated these influences. Based on the comparison results of nested family SES and age moderated models of organized activity involvement, and considering both the chi-square difference tests and AIC, the model with family SES × age moderation for E component, but not for the AC components, provided the best fit to the data. Table 6 presents the best-fitting model decomposing organized activity involvement with moderation by family SES and age. There was no evidence that age moderated significantly the influence of family SES on the A component. These results indicate that the High-SES Amplification hypothesis, that the A component is positively moderated by family SES, is supported across ages 4–25.

**Table 6:** Decomposition of organized activity involvement into ACE components by family SES and age.

Parameter	Model 1
A	0.406***
	(0.020)
C	0.683***
	(0.017)
E	0.424***
	(0.007)
Sex	−0.035
	(0.028)
Family SES	0.269***
	(0.014)
Age	−0.185***
	(0.015)
Family SES × age	−0.051***
	(0.014)
A × family SES	0.059***
	(0.013)
A × age	0.110***
	(0.015)
C × age	−0.231***
	(0.018)
E × family SES	0.015*
	(0.006)
E × age	0.193***
	(0.006)
E × family SES × age	0.019***
	(0.005)

Standard errors are in parentheses; *p < 0.05, **p < 0.01, ***p < 0.001; organized activity involvement, family SES, and age are standardized; sex (0 = male, 1 = female); intercept is omitted; N twins = 7,987 and N families = 4,022. Data source: German Twin Family Panel (TwinLife).

## 4 Discussion

This study examined how family socioeconomic status (SES) shapes the realization of genetic potential in organized activity involvement. Using data from the German Twin Family Panel (TwinLife) and classical twin models, we found that genetic influences positively contribute to organized activity involvement across all SES levels. Consistent with the High-SES Amplification hypothesis, higher-SES contexts show stronger genetic influences. These findings show that more advantaged environments are associated with greater expression of genetic potential in organized activity involvement.

These results advance understanding of inequality in several ways. First, they identify a concrete mechanism linking family SES to disparities in organized activity involvement: family resources amplify the realization of genetic potential. This provides a biosocial explanation for gaps in organized activity involvement across social groups. The pattern observed for organized activity involvement aligns with previous research on the realization of genetic potential in other domains, such as intellectual development in the United States (Guo and Stearns, 2002; Tucker-Drob and Bates, 2016). Moreover, the realization of genetic potential in outcomes such as intellectual development and educational achievement varies across countries (Tucker-Drob and Bates, 2016; Baier et al., 2022). Evidence from both within-society studies, such as the present analysis, and cross-national research demonstrates that social contexts substantively shape the realization of genetic potential across multiple domains and underscores the importance of integrating behavioral genetics and sociological perspectives in the study of inequality.

Several limitations and concerns should be noted. First, the classical twin design has inherent limitations. For example, this design cannot model passive gene–environment correlation (rGE), in which a genetic propensity for a phenotype may be correlated with the provision of phenotype-enhancing rearing environments (Plomin et al., 1977), and estimation of genetic and environmental influences may be biased (Coventry and Keller, 2005; Keller et al., 2010; Verhulst and Hatemi, 2013). Further research could apply designs such as the nuclear twin family design (Keller et al., 2010), an extension of the classical twin design, to provide additional evidence on the role of passive rGE in organized activity involvement.

Second, regarding concerns about measurement comparability across birth cohorts, although several activity types were cohort-specific, the outcome was operationalized as overall monthly frequency of organized activity involvement, and all cohorts included an “other organized activities” category. Thus, from a frequency-based perspective, the construct was assessed comparably across cohorts. The cohort-specific items primarily reflect developmentally appropriate differences in available activity types rather than differences in the construct of organized involvement. At the same time, the activities assessed span multiple domains, including leisure, social, and civic categories, and the relative contributions of genetic and environmental factors may differ across these domains given their distinct social mechanisms. Future research could examine domain-specific patterns of genetic and environmental influence across activity types and developmental stages.

Third, we assigned a value of 0.5 to the category “less than once a month” of organized activity involvement to approximate overall monthly participation frequency across a broad range of activities. This is arbitrary and represents a limitation of the current operationalization. We did not choose the response options as an ordinal scale (e.g., 0 = never, 1 = less than once a month, 2 = every month, 3 = every week), because the index aggregates participation across different activities; such coding would make it more difficult to construct an interpretable indicator of overall participation frequency that is comparable across age groups. Therefore, while we acknowledge this limitation, we retained the current indicator because it provides a pragmatic approximation of overall participation frequency across activities and age groups.

Fourth, family SES in this study is a relative measure of advantage and disadvantage within a single society. Further research could examine the specific opportunities and constraints associated with SES. Fourth, the sample is representative of Germany; therefore, the findings may not generalize to countries with different social contexts.

Despite these limitations and concerns, the present study makes two contributions. First, it identifies a concrete mechanism underlying family SES disparities in organized activity involvement by demonstrating how family resources shape the realization of genetic potential using representative national data. Second, it advances interdisciplinary scholarship by integrating sociological theories of stratification with behavioral genetic approaches to gene–environment interaction.

In summary, this study demonstrates that high family SES amplifies the influence of genetic differences on organized activity involvement, highlighting the critical role of family-level opportunities and constraints in enabling the realization of individual genetic potential. By integrating insights from behavioral genetics and sociology, we show that social context shapes the realization of genetic potential and contributes to socioeconomic disparities in organized activity involvement. These findings highlight the relevance of environmental conditions for supporting individuals in realizing their genetic potential in social life.

## Data Availability

Publicly available datasets were analyzed in this study. This data can be found here: the TwinLife data are archived in the GESIS data catalog: https://search.gesis.org/research_data/ZA6701. Data is released for academic research and teaching after the data depositor’s written authorization.
