# Supplementary material for: Family SES amplifies genetic influences on organized activity involvement from childhood to young adulthood: evidence from the German twin family panel
Source: Front Sociol. 2026 Jun 30;11:1780253. doi: 10.3389/fsoc.2026.1780253 (PMC13363328; doi:10.3389/fsoc.2026.1780253)
Supplement: Supplementary file 1 [file Table_1.DOCX]

# 1. Supplementary materials

s[Table 1](#tbl-check_corr_cgr) displays the results of the correlation between the organized activity involvement of twins by birth cohort and zygosity.

| sTable 1: Correlation between the organized activity involvement of twins by birth cohort and zygosity   \| Group \| Parameter1 \| Parameter2 \| r \| 95% CI \| t \| p \| N \| \| --- \| --- \| --- \| --- \| --- \| --- \| --- \| --- \| \| 1 - 1 \| OAI_1 \| OAI_2 \| 0.98 \| [0.97, 0.98] \| 126.44 \| 0.000 \| 832 \| \| 2 - 1 \| OAI_1 \| OAI_2 \| 0.91 \| [0.90, 0.92] \| 62.58 \| 0.000 \| 836 \| \| 3 - 1 \| OAI_1 \| OAI_2 \| 0.67 \| [0.63, 0.70] \| 28.26 \| 0.000 \| 994 \| \| 4 - 1 \| OAI_1 \| OAI_2 \| 0.58 \| [0.54, 0.62] \| 23.25 \| 0.000 \| 1050 \| \| 1 - 2 \| OAI_1 \| OAI_2 \| 0.94 \| [0.94, 0.95] \| 93.30 \| 0.000 \| 1088 \| \| 2 - 2 \| OAI_1 \| OAI_2 \| 0.77 \| [0.75, 0.79] \| 41.30 \| 0.000 \| 1172 \| \| 3 - 2 \| OAI_1 \| OAI_2 \| 0.48 \| [0.43, 0.52] \| 17.86 \| 0.000 \| 1078 \| \| 4 - 2 \| OAI_1 \| OAI_2 \| 0.25 \| [0.19, 0.32] \| 7.74 \| 0.000 \| 866 \| |
| --- | --- | --- | --- | --- | --- | --- | --- | --- | --- | --- | --- | --- | --- | --- | --- | --- | --- | --- | --- | --- | --- | --- | --- | --- | --- | --- | --- | --- | --- | --- | --- | --- | --- | --- | --- | --- | --- | --- | --- | --- | --- | --- | --- | --- | --- | --- | --- | --- | --- | --- | --- | --- | --- | --- | --- | --- | --- | --- | --- | --- | --- | --- | --- | --- | --- | --- | --- | --- | --- | --- | --- | --- |

Note. Organized activity involvement (OAI) is adjusted for age and sex when calculating correlations. OAI_1 and OAI_2 refer to the first and second twin in each pair, respectively. Groups 1–4 represent birth cohorts 1–4. Groups labeled “–1” refer to monozygotic (MZ) twins, and groups labeled “–2” refer to dizygotic (DZ) twins.

s[Table 2](#tbl-comt1) displays the results of the comparison of ACE, AE, CE, and E models based on chi-square difference tests and AIC. Based on these results, all nested models of the ACE model showed significantly worse fit, indicating that the ACE model provided the best fit to the data.

| sTable 2: Comparison of ACE, AE, CE, and E models on organized activity involvement   \| Expanded model \| Nested model \| AIC (nested) \| Δ-2LL \| Δdf \| p \| \| --- \| --- \| --- \| --- \| --- \| --- \| \| ACE \| CE \| 19200 \| 75.24 \| 1 \| 0.000 \| \| ACE \| AE \| 19509 \| 383.41 \| 1 \| 0.000 \| \| AE \| E \| 22273 \| 2766.73 \| 1 \| 0.000 \| \| CE \| E \| 22273 \| 3074.90 \| 1 \| 0.000 \| \| ACE \| E \| 22273 \| 3150.14 \| 2 \| 0.000 \| |
| --- | --- | --- | --- | --- | --- | --- | --- | --- | --- | --- | --- | --- | --- | --- | --- | --- | --- | --- | --- | --- | --- | --- | --- | --- | --- | --- | --- | --- | --- | --- | --- | --- | --- | --- | --- | --- |

Note. Organized activity involvement was modeled using univariate variance decomposition models. All models adjust for sex and age. ACE represents additive genetic (A), shared environmental (C), and non-shared environmental (E) components. AE, CE, and E models are nested submodels of the ACE model. Model comparisons were conducted using chi-square difference tests (Δ−2LL), with corresponding differences in degrees of freedom (Δdf). A significant Δ−2LL indicates that the nested model provides a significantly poorer fit relative to the expanded model. Non-nested models were compared using AIC, with lower values indicating better fit.

s[Table 3](#tbl-comt2) displays the comparison results of nested moderated models based on chi-square difference tests and AIC. Considering both the chi-square difference tests and AIC, the model with A × family SES and C × family SES (ACEMMAMCM) provided the best fit to the data.

| sTable 3: Comparison of nested moderated models on organized activity involvement   \| Expanded model \| Nested model \| AIC (nested) \| Δ-2LL \| Δdf \| p \| \| --- \| --- \| --- \| --- \| --- \| --- \| \| ACEMMAMCMEM \| ACEMMAMCM \| 18749 \| 2.2256 \| 1 \| 0.136 \| \| ACEMMAMCMEM \| ACEMMAMEM \| 18750 \| 4.1609 \| 1 \| 0.041 \| \| ACEMMCMEM \| ACEMMCM \| 18751 \| 0.0002 \| 1 \| 0.988 \| \| ACEMMAMCM \| ACEMMCM \| 18751 \| 4.6844 \| 1 \| 0.030 \| \| ACEMMAMCMEM \| ACEMMCM \| 18751 \| 6.9100 \| 2 \| 0.032 \| \| ACEMMAMCMEM \| ACEMMCMEM \| 18753 \| 6.9098 \| 1 \| 0.009 \| \| ACEMMAMEM \| ACEMMAM \| 18753 \| 4.7173 \| 1 \| 0.030 \| \| ACEMMAMCM \| ACEMMAM \| 18753 \| 6.6525 \| 1 \| 0.010 \| \| ACEMMAMCMEM \| ACEMMAM \| 18753 \| 8.8782 \| 2 \| 0.012 \| \| ACEMMEM \| ACEMM \| 18777 \| 0.6307 \| 1 \| 0.427 \| \| ACEMMAM \| ACEMM \| 18777 \| 25.9368 \| 1 \| 0.000 \| \| ACEMMCM \| ACEMM \| 18777 \| 27.9050 \| 1 \| 0.000 \| \| ACEMMCMEM \| ACEMM \| 18777 \| 27.9052 \| 2 \| 0.000 \| \| ACEMMAMEM \| ACEMM \| 18777 \| 30.6541 \| 2 \| 0.000 \| \| ACEMMAMCM \| ACEMM \| 18777 \| 32.5894 \| 2 \| 0.000 \| \| ACEMMAMCMEM \| ACEMM \| 18777 \| 34.8150 \| 3 \| 0.000 \| \| ACEMMCMEM \| ACEMMEM \| 18778 \| 27.2745 \| 1 \| 0.000 \| \| ACEMMAMEM \| ACEMMEM \| 18778 \| 30.0234 \| 1 \| 0.000 \| \| ACEMMAMCMEM \| ACEMMEM \| 18778 \| 34.1843 \| 2 \| 0.000 \| \| ACEMM \| ACE \| 19127 \| 352.0373 \| 1 \| 0.000 \| \| ACEMMEM \| ACE \| 19127 \| 352.6680 \| 2 \| 0.000 \| \| ACEMMAM \| ACE \| 19127 \| 377.9741 \| 2 \| 0.000 \| \| ACEMMCM \| ACE \| 19127 \| 379.9423 \| 2 \| 0.000 \| \| ACEMMCMEM \| ACE \| 19127 \| 379.9425 \| 3 \| 0.000 \| \| ACEMMAMEM \| ACE \| 19127 \| 382.6914 \| 3 \| 0.000 \| \| ACEMMAMCM \| ACE \| 19127 \| 384.6266 \| 3 \| 0.000 \| \| ACEMMAMCMEM \| ACE \| 19127 \| 386.8523 \| 4 \| 0.000 \| |
| --- | --- | --- | --- | --- | --- | --- | --- | --- | --- | --- | --- | --- | --- | --- | --- | --- | --- | --- | --- | --- | --- | --- | --- | --- | --- | --- | --- | --- | --- | --- | --- | --- | --- | --- | --- | --- | --- | --- | --- | --- | --- | --- | --- | --- | --- | --- | --- | --- | --- | --- | --- | --- | --- | --- | --- | --- | --- | --- | --- | --- | --- | --- | --- | --- | --- | --- | --- | --- | --- | --- | --- | --- | --- | --- | --- | --- | --- | --- | --- | --- | --- | --- | --- | --- | --- | --- | --- | --- | --- | --- | --- | --- | --- | --- | --- | --- | --- | --- | --- | --- | --- | --- | --- | --- | --- | --- | --- | --- | --- | --- | --- | --- | --- | --- | --- | --- | --- | --- | --- | --- | --- | --- | --- | --- | --- | --- | --- | --- | --- | --- | --- | --- | --- | --- | --- | --- | --- | --- | --- | --- | --- | --- | --- | --- | --- | --- | --- | --- | --- | --- | --- | --- | --- | --- | --- | --- | --- | --- | --- | --- | --- | --- | --- | --- | --- | --- | --- | --- |

Note. Organized activity involvement was modeled using moderated univariate variance decomposition models. All models adjust for sex and age. ACE represents additive genetic (A), shared environmental (C), and non-shared environmental (E) influences. MM refers to moderation of the mean by family SES. AM, CM, and EM refer to moderation of the additive genetic (A), shared environmental (C), and non-shared environmental (E) components by family SES. Model comparisons were conducted using chi-square difference tests (Δ−2LL), with corresponding differences in degrees of freedom (Δdf). A significant Δ−2LL indicates that the nested model provides a significantly poorer fit relative to the expanded model. Non-nested models were compared using AIC, with lower values indicating better fit.
